# Novel Multiparametric Bioelectronic Measurement System for Monitoring Virus-Induced Alterations in Functional Neuronal Networks

**DOI:** 10.3390/bios14060295

**Published:** 2024-06-05

**Authors:** Heinz-Georg Jahnke, Verena te Kamp, Christoph Prönnecke, Sabine Schmidt, Ronny Azendorf, Barbara Klupp, Andrea A. Robitzki, Stefan Finke

**Affiliations:** 1 Centre for Biotechnology and Biomedicine, Biochemical Cell Technology, Leipzig University, Deutscher Platz 5, 04103 Leipzig, Germanysabine.schmidt@bbz.uni-leipzig.de (S.S.);; 2 Institute of Molecular Virology and Cell Biology, Friedrich-Loeffler-Institut, Südufer 10, 17493 Greifswald, Germanybarbara.klupp@fli.de (B.K.);; 3 Division Management for Biology, Chemistry and Process Engineering, Karlsruhe Institute of Technology (KIT), Hermann-von-Helmholtz-Platz 1, 76344 Eggenstein-Leopoldshafen, Germany

**Keywords:** impedance spectroscopy, field potential monitoring, microelectrode arrays, primary hippocampal neurons, pseudorabies virus model

## Abstract

Development and optimisation of bioelectronic monitoring techniques like microelectrode array-based field potential measurement and impedance spectroscopy for the functional, label-free and non-invasive monitoring of in vitro neuronal networks is widely investigated in the field of biosensors. Thus, these techniques were individually used to demonstrate the capabilities of, e.g., detecting compound-induced toxicity in neuronal culture models. In contrast, extended application for investigating the effects of central nervous system infecting viruses are rarely described. In this context, we wanted to analyse the effect of herpesviruses on functional neuronal networks. Therefore, we developed a unique hybrid bioelectronic monitoring platform that allows for performing field potential monitoring and impedance spectroscopy on the same microelectrode. In the first step, a neuronal culture model based on primary hippocampal cells from neonatal rats was established with reproducible and stable synchronised electrophysiological network activity after 21 days of cultivation on microelectrode arrays. For a proof of concept, the pseudorabies model virus PrV Kaplan-ΔgG-GFP was applied and the effect on the neuronal networks was monitored by impedance spectroscopy and field potential measurement for 72 h in a multiparametric mode. Analysis of several bioelectronic parameters revealed a virus concentration-dependent degeneration of the neuronal network within 24–48 h, with a significant early change in electrophysiological activity, subsequently leading to a loss of activity and network synchronicity. In conclusion, we successfully developed a microelectrode array-based hybrid bioelectronic measurement platform for quantitative monitoring of pathologic effects of a herpesvirus on electrophysiological active neuronal networks.

## 1. Introduction

Investigation of viruses in their hosts with regard to pathological effects has always been at the core focus of research since viruses were discovered. Even though most viruses simply use host cells to replicate themselves and then destroy the host cells, there are many known virus-induced effects that subtly alter cellular properties and processes like induction of mitophagy [1], alteration of host miRNA expression [2] or epigenotype, as well as gene expression pattern [3]. Moreover, all behaviour of the host could be transformed, like a decrease in cognitive functioning [4]. For example, lentiviral expression of the rabies virus glycoprotein altered the neuronal network structure and even enhanced spatial memory [5], suggesting that the neurotropic rabies virus is able to infect the central and peripheral neurons [6] and is thus able to modify neuronal functionality through specific interactions. In humans, rabies encephalitis is accompanied by symptoms like hallucinations, aggressive behaviour, strong sweating and salivation, hydrophobia or even an addiction to bite [7], further suggesting that neuronal functions and communication are affected. Despite these phenomenological effects in the virus, hosts are widely known and investigated, while the exact mechanisms on a (sub)cellular level in neurons and the central nervous system are poorly understood. Therefore, an actual focus of research in this field is the development of in vitro models for investigating molecular virology mechanisms. In this context, the swine herpesvirus (PrV) is a very interesting model for virology and neurology because it is able to infect synaptically connected neurons and, therefore, provides a great opportunity for investigating neuroinvasion from attachment and entry to replication, intracellular trafficking and egress, transneuronal spread and immune responses of the host [8,9]. Now that in vitro model systems are getting better and better, and the biological toolbox for mechanism analysis continuously expands, there is a high demand for functional monitoring technologies, especially for in vitro models of neuronal networks. In this context, microelectrode-based field potential monitoring is perfectly suited for the spatial resolved analysis of electrophysiological network activity [10,11,12]. Another appropriate method is impedance spectroscopy, which is highly suited for monitoring morphological changes and cellular degeneration [13,14,15]. The combined use of these label-free, non-invasive monitoring techniques would allow for a comprehensive multimodal analysis of complex virus-induced effects in neuronal networks. While hybrid measurement systems are already available in the field of cardiomyocyte models [16], these systems are only based on individual large-area electrodes for the impedimetric measurement and separated microelectrodes for the field potential monitoring. Due to the technical challenges, systems that offer high-resolution impedance spectroscopy, as well as field potential monitoring on the same microelectrode while also comprising a sufficient number of electrodes per culture unit, are still not yet described or even commercially available. In this context, our aim was to develop a hybrid, i.e., multimodal and multiparametric measurement system for combined high-resolution impedance spectroscopy and field potential monitoring on microelectrode arrays for spatial resolved high-content analysis of neuronal networks. For a proof of concept, we used the pseudorabies model virus PrV Kaplan-ΔgG-GFP on rat primary hippocampal networks for analysing the effect of this virus in detail.

## 2. Materials and Methods

### 2.1. Fabrication of Microelectrode Arrays

Microelectrode arrays were produced in our clean room facility using the lift-off technique as previously described in [10]. Briefly, glass substrates were cleaned and structured with photoresist (AR-P 3510, Allresist GmbH, Strausberg, Germany) using a Mask Aligner (MA6, SÜSS MicroTec GmbH, Garching, Germany) to form conducting paths and electrodes. A 350 nm thick gold coating was applied by a sputtering process in a CREAMET 500 high vacuum device (CREAVAC GmbH, Dresden, Germany). The passivation layer was fabricated using the photoresist SU8-2 (KAYAKU Advanced Materials, Newton, MA, USA). For final cleaning and hydrophilisation, microelectrode arrays were plasma treated in an argon atmosphere with 400 mA for 450 s (CREAMET 500, CREAVAC GmbH, Dresden, Germany).

### 2.2. Bioelectronic Measurement System

For the development of the hybrid measurement setup, a specific adapted data acquisition (DAQ) system was obtained from Sciospec Scientific GmbH (Bennewitz, Germany). Briefly, the system comprised two 96-channel data acquisition cards with an integrated 200× amplifier and a low pass filter. Furthermore, a high-precision impedance analyser module was integrated. For automated measurement, LabView 2018 (National Instruments)-based software was developed for impedance measurement (IMATadvanced v1) and field potential monitoring (FiPRAT v1). Impedance spectra were recorded from 500 Hz to 5 MHz (51 frequency points), with a signal amplitude of ±10 mV. Field potential streams were recorded with 20 kHz for at least 6 min for each time point.

### 2.3. Primary Neuronal Cell Culture and Virus Used for Infection

All performed work described in this article has been carried out in accordance with the German legal and ethical requirements of appropriate procedures. All animal work followed national and international guidelines in accordance with Directive 86/609/EEC for animal experiments in and under the responsibility of the Friedrich-Loeffler-Institut. One day old Spraque Dawley rats from our own animal house (hosted by the Friedrich-Loeffler-Institut) were decapitated; the skull was opened and the brain was transferred to an HBSS (Life Technologies, Carlsbad, CA, USA)-containing petri dish. The cerebellum and hypothalamus were removed and the hemispheres were divided. The midbrain of each hemisphere was removed; the hippocampus was separated and mechanically minced with a scalpel. The hippocampi were transferred to a 15 mL tube with 10 mL ice cold HBSS (two hippocampi per tube) and centrifuged (2 min, 200× *g*, 4 °C). The supernatant was removed and 1 mL 0.25% trypsin/EDTA (Life Technologies), as well as 0.5 mL DNase I (Applichem, Darmstadt, Germany), was added, followed by incubation at 37 °C for 30 min under rotation (150 rpm). The cells were carefully resuspended in 2 mL NB-A medium (Life Technologies) containing 10% foetal calf serum (Life Technologies) using a fire-rounded glass pipette and centrifuged for 5 min at 500× *g* and 4 °C. The cells were resuspended in NB-A medium without supplements, combined and counted using a LUNA automated cell counter (Logos Biosystems, Villeneuve, France). Cells were seeded on Matrigel™ (Corning Inc., Corning, NY, USA) pre-coated (1:20 dilution, 3 h at room temperature) microelectrode arrays (250,000 cells/MEA). Medium was changed the next day and half of the medium was changed every second day thereafter.

For infection of neuron cultures, a previously described recombinant GFP expressing PrV Kaplan-ΔgG-GFP with the non-essential gG gene deleted was used [17].

### 2.4. Data Analysis and Statistics

Impedance spectra data were analysed using a self-developed software (IDAT v4, LabView), which calculates the cellular contribution from the impedance magnitude spectra ((|Z|cell − |Z|cellfree)/|Z|cell-free × 100%), and extracts the maximum value for each spectrum and traces it over time. The electrical equivalent circuit model fitting is based on a non-linear optimisation function with constraints provided by LabView. Determination of the cellular parameters was achieved using a two-step fitting procedure. In the first step, blank impedance spectra (without cells) were used to determine the system parameters. In the second step, these determined system parameters were set as the constant for fitting the cell parameters.

Recorded field potential streams were analysed using a self-developed software (FiPRAT, LabView). First, action potentials were detected by an automated threshold-based algorithm. In detail, the basic noise level is dynamically determined based on a median analysis as previously described [18]. Instead of a multiplication factor of four for getting the absolute threshold level, we used six for a safe exclusion of false positive spikes. Based on the detected action potentials, descriptive parameters were analysed within intervals of 5 min for each time point. Network synchronicity was analysed by spike population coherence [19] with a time constant (tau) of 50 ms. GraphPad Prism 9 was used for all statistical analyses. To test for significant effects between multiple groups, an ANOVA test with a Tukey post hoc test (recommended by GraphPad Prism) was performed.

## 3. Results and Discussion

### 3.1. Development of the Hybrid Bioelectronic Measurement System

**Figure 1:**
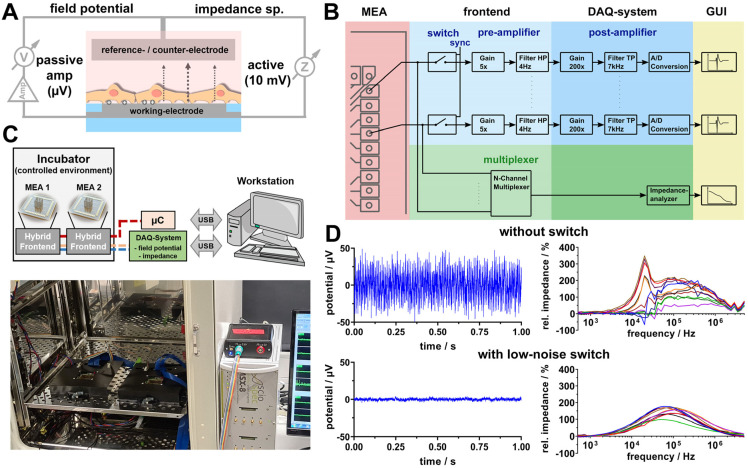
Description of hybrid measurement system. (**A**) Scheme of combined field potential and impedance spectroscopy measurement on the same microelectrode. (**B**) Scheme of field potential circuit pathway and high-precision impedance spectroscopy pathway based on channel multiplexing. Connection of both pathways on the measurement electrode is realised by a low noise switch. (**C**) Scheme and photo of whole measurement setup. (**D**) Measurement signals (field potential baseline and cell signal represented by relative impedance) of directly connected (without switch) measurement pathways and pathways connected over low noise switches.

For the comprehensive, spatially resolved, label-free bioelectronic analysis of neuronal networks, microelectrode arrays are a prerequisite. Furthermore, the combination of different bioelectronic monitoring techniques like field potential measurement and impedance spectroscopy are excellently suited for spatially resolving electrophysiological activity, as well as cellular morphology and the viability of neuronal in vitro cultures. While both techniques are widely used individually, the concept of using both techniques on the same microelectrode (Figure 1A) has not been satisfactorily demonstrated for real-time measurement applications thus far. One major reason for this is the opposite nature of these techniques with, on the one hand, an amplification and detection of weak extracellular potentials in the µV range, and on the other hand, an active measurement where signals in the range of mV are applied for the frequency-dependent analysis of the response.

Moreover, using microelectrodes, which have a high intrinsic impedance, poses major challenges on the measurement equipment for each technique, like the high-precision impedance analyser, as well as all connecting and multiplexing circuits. In this context, we developed a measurement path for each technique that could be integrated in a single frontend for the connection of microelectrode arrays (Figure 1B). First experiments with both measurement paths connected directly to the same electrode revealed clear interreferences for both measurement techniques, even when they were performed in sequence (Figure 1D). Thus, the field potential baseline of microelectrodes (100 µm diameter) showed maximum signals of up to 40 µV and a noise level (standard deviation) of >15 µV, which is definitely too high for a sensitive detection of neuronal action potentials [10]. In accordance therewith, high noise levels were observed within a wide frequency range of impedance spectra and, thereof, derived frequency-dependent cell signals (Figure 1D). In the search for a solution, we investigated several switch circuits for isolating both measurement pathways from each other, which, in fact, was quite difficult because those circuits also easily induce noise, especially in the field potential amplifier pathway. Finally, we were able to identify the low noise switch circuit ADG1212 from analog devices that offer a low on (1 pF)/off (2.6 pF) capacitance, low charge injection (1 pC) and low channel-to-channel crosstalk of 90 dB, resulting in minimum noise and measurement signal disturbances. These switches were introduced for each channel in front of both measurement pathways (measurement electrodes) and synchronised by a microcontroller (Figure 1B). These switches were also introduced for switching between reference electrode (field potential) and counter-electrode (impedance) paths. Based on this circuit design, a hybrid frontend that provides a field potential baseline of microelectrodes (100 µm diameter) with maximum signals < 3 µV and a noise level (standard deviation) of 1.8 µV (Figure 1D) was realised. For the realisation of a complete measurement setup, the hybrid frontend was designed with the aim for use in a controlled environment for cell culture (incubator). With respect to a stable temperature of 37 °C during continuous operation, the heat dissipation by introduced energy was in focus. Therefore, the 60-channel field potential amplifier was divided into two stages, where only the first stage was integrated into the frontend (amplification factor of 5) and the second stage (amplification factor of 200) was integrated into the custom-made DAQ system obtained from Sciospec Scientific Instruments. Based on this approach, the whole energy consumption of the frontend could be limited to 2.1 W. Using a standard commercially available cell incubator, two frontends could be placed in parallel for an increased measurement throughput. The frontends are connected by flat ribbon cable to the DAQ system and to a microcontroller box outside of the incubator. The DAQ system and microcontroller box are connected by USB cable to the workstation (Figure 1C). For performing automated measurements with both measurement techniques on both frontends in parallel, controlling software was developed for field potential monitoring (FiPRAT) and impedance spectroscopy (IMATadvanced) (Supplementary Figure S1)

### 3.2. Establishment of Primary Hippocampal Culture Model on Optimised Microelectrode Arrays

**Figure 2:**
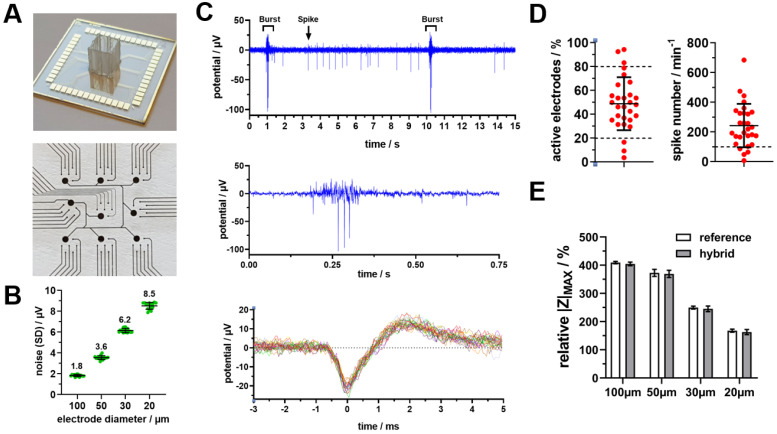
Microelectrode size optimisation and bioelectronic characterisation of primary hippocampal cultures. (**A**) Images of used microelectrode arrays with 54 measurement electrodes and a shared reference/counter-electrode with a bonded 8.2 mm × 8.2 mm culture chamber. (**B**) Noise (standard deviation) analysis for field potential base signal of microelectrodes with different diameter (n = 18 electrodes, mean ± sd). (**C**) Representative field potential signal of electrophysiological active rat hippocampal culture (day 21) at different time scales, demonstrating different signal patterns (single spikes and spike clusters called bursts). High-resolution single spikes are shown as an overlay of 30 spikes from a single electrode. (**D**) Basic characterisation of electrophysiological activity for defining quality criteria of used cultures in experiments. Dashed lines represent minimum and maximum criteria (n = 28, mean ± sd). (**E**) Maximum relative impedance (cell signal) for only an impedance reference frontend in comparison to the novel hybrid frontend (n = 8, mean ± sd).

In addition to the measurement setup, the microelectrode arrays are the most crucial factor for a robust and sensitive monitoring. Therefore, we started with a microelectrode layout that was successfully used in a previous study for the characterisation of human-induced pluripotent stem cell (hiPSC)-derived neuronal network maturation [10]. This array comprises 54 measurement microelectrodes with a diameter of 50 µm on a cultivation area of 0.67 cm^2^ and a reference electrode with a total area of 1.77 mm^2^ (Figure 2A). The size of the microelectrode was originally adapted for detecting action potentials with a signal amplitude of at least 15 µV, which was successfully used for the performed analysis and comparison of hiPSC-derived neuronal networks.

Since our new hybrid system offers an excellent low basic DAQ noise characteristic and consists of two different measurement techniques, we investigated in detail the influence of the electrode size. First, the amount of detectable action-potential-derived field potential spikes was analysed in depth for electrophysiological active primary hippocampal cultures. Therefore, microelectrode arrays with electrode diameters of 20 µm, 30 µm, 50 µm and 100 µm were fabricated. Regarding the reference/counter to the working electrode area ratio, even the largest measurement microelectrode (100 µm) leads to a ratio of 225, which ensures that the working electrode is the dominant electrode in the impedance measurement and that the reference electrode provides a stable reference potential for the field potential monitoring when connected to the technical ground. The determined basic noise levels were as follows (Figure 2B): the lowest was at 1.8 µV for 100 µm and twice as high at 3.6 µV for 50 µm, 6.2 µV for 30 µm and 8.5 µV for 20 µm. Based on this, we recorded field potential streams from electrophysiological active primary hippocampal cultures and analysed the number of detected action potentials (spikes) depending on the basic noise level, on which the detection threshold is calculated (Supplementary Figure S2). Astonishingly, doubling the basic noise level from 1.8 µV (100 µm) to 3.6 µV (50 µm) led to a reduction of recognised spikes by 90%. Additionally, we analysed the impedance for the selected electrode sizes in comparison to a reference frontend that was developed and optimized for analysis based on only impedance spectroscopy (Supplementary Figure S3 and Figure 2E). The analysis revealed no deviations in the impedance magnitude spectra as well as for the maximum achievable cell signals. Furthermore, a smaller electrode size correlates with decreased cell signals for both systems. This is in line with a previous study, where signals of HEK-293A cells were analysed for electrodes with a diameter between 50 µm and 200 µm [20], with a maximum signal amplitude for 100 µm large electrodes. Although smaller electrode sizes are in general favoured for the analysis of neuronal cultures to achieve higher specificity and separation of individual neuronal signals, we have chosen the 100 µm large electrodes with respect to the significant higher number of detectable action potentials, as well as higher impedimetric cell signal amplitudes. Thus, we fabricated new microelectrode arrays (MEA) with an electrode size of 100 µm and characterised the field potential signals from hippocampal cultures demonstrating detection of activity patterns (single spikes and bursts), including high-resolution of a single spike with a duration of 1–3 ms (Figure 2C) as well as achievable overall activity in detail (Figure 2D). First, we analysed several preparations regarding the time point for starting the experiments. Although the first electrophysiological activity was in general observable within the first two weeks, at least three weeks were needed for the establishment of robust spontaneous activity with high numbers of spike and burst signals (Figure 2C). Since biological variations within different preparations and between different MEAs naturally occur for the electrophysiological activity, we defined quality criteria for the use in experiments in the next step. Based on 28 MEAs with cells from four different preparations and the ability to observe virus-induced alterations in both directions, we set a lower and upper limit of 20% and 80% for the number of active electrodes and 100 spikes per minute (Figure 2D).

### 3.3. Impedance Spectroscopy-Based Monitoring of Virus-Induced Degeneration of the Neuronal Networks

**Figure 3:**
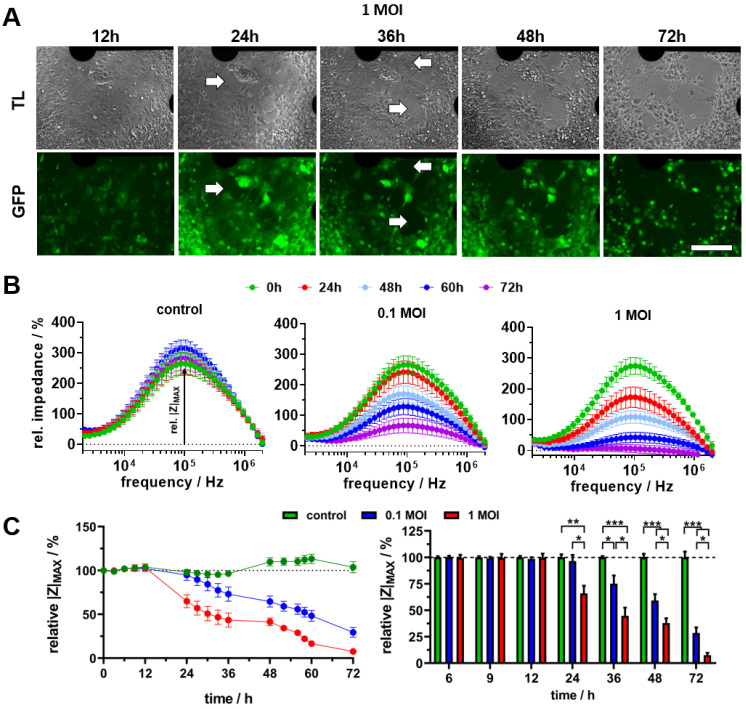
Virus-induced morphological degeneration of the neuronal network. (**A**) Transmitted light (TL) and fluorescence (GFP) microscopic images of neuronal cultures on an exemplarily MEA incubated with 1 MOI virus at discrete time points. White arrows mark initial areas of morphological dissolution/degeneration of the cell layer (scale bar = 200 µm). (**B**) Determined cellular contribution to impedance magnitude spectra (relative impedance) at selected time points (n = 6, mean ± sem). (**C**) Relative impedance spectra-derived maximum cell signal (relative impedance maximum) traces of control, 0.1 MOI and 1 MOI virus normalised to time point 0 h (**left**) as well as statistical analysis of values normalised to control for selected time points (**right**). (n = 6, mean ± sem, * *p* < 0.05, ** *p* < 0.01, *** *p* < 0.001).

For a proof of concept, the pseudorabies model virus PrV Kaplan-ΔgG-GFP was used for investigating the effect on the established hippocampal neurons cultured on MEAs. Due to the GFP label introduced into this virus, the infection of the cultures could be easily proved and monitored over the whole time of the experiment (Supplementary Figure S4). For the analysis of the virus-amount-dependent effect, two concentrations (multiplicity of infection—MOI) were applied on day 21 after cell-seeding on MEAs, followed by 72 h of bioelectronic and microscopic monitoring of the virus-induced effects. The successful infection could be observed already after 12 h. While for an MOI of 0.1 this was generally observed in single cells, a widespread infection could be observed for an MOI of 1 (Supplementary Figures S4 and S5 and Figure 3A). During the following 24 h, the level of infected cells resembled each other. In combination with the transmitted light images, the morphological degeneration of the neuronal networks could be clearly observed. For an MOI of 1, first dissolution of the cell layer could be observed already after 24 h (Figure 3A), whereas for an MOI of 0.1, this was clearly visible only after 48 h (Supplementary Figure S5). After 72 h, a comprehensive dissolution of the whole cell layer was observable for both concentrations.

For bioelectronic monitoring of this degeneration process, we used impedance spectroscopy, which is widely used for in vitro analyses of cell degeneration and cytotoxic effects [14,21], in contrast to field potential monitoring.

For a comprehensive statistical analysis, we monitored six MEAs per group with cells from three different preparations (n = 6). The extracted cellular contribution to the impedance magnitude spectra (relative impedance spectra) revealed the high stability of the established neuronal cell cultures over the entire experimental time period of three days, as well as the time- and virus-amount-dependent cell layer degenerating effect (Figure 3B). From the relative impedance spectra of each electrode, the maximum cell signal was determined and traced over time (Figure 3C, left). The statistical analysis of the averaged time traces confirmed the high stability of the control cultures due to the small deviation of mainly ± 5%, except for a slight increase up to 113% between 48 h and 60 h, which is still a small change. In contrast, the virus application led to a concentration-dependent continuous decrease in the relative impedance after 24 h. For a statistical analysis of significant effects, values of selected time points were normalised to the appropriate control traces (Figure 3C, right). Thus, a significant decrease could be observed for an MOI of 1, beginning after 24 h (65.9 ± 7.2 %) and resulting in a decrease down to 7.5 ± 2.1 % after 72 h. For an MOI of 0.1, a first significant decrease was observed after 36 h (75.1 ± 7.7 %), followed by a continuous decrease down to 28.5 ± 5.3 % after 72 h. This observed time-dependent decrease in the impedimetric cell signal perfectly correlates with the microscopically observed degeneration of the cell layer. Additionally, we performed an electrical equivalent circuit-model-based fitting of the complex impedance dataset to receive more insights in the observed effects (Figure S6). The fitting of spectra from cell cultures could be successfully performed with a capacitance (C_Cell_) and resistance (R_Para_), parallel to an additional resistance in the series (R_Ser_) (Figure S6A,B). The statistical analysis of these biological parameters revealed a virus-amount- and time-dependent increase in C_Para_, as well as a decrease in R_Ser_ (Figure S6C,D). This proves the suitability of impedance spectroscopy as a monitoring technique for degeneration of cell layers and cytotoxic effects and correlates with previous studies of impedance spectroscopy-based neurodegeneration monitoring on neuronal cell lines [13,15,22]. In this context, the decrease of up to 92% means the extreme degeneration and damage of the whole cell layer, at least for the higher virus concentration. This is in line with previous studies using PrV Kaplan-ΔG-GFP [23].

### 3.4. Field-Potential-Based Analysis of Virus Effects on the Electrophysiological Activity of Neural Networks

**Figure 4:**
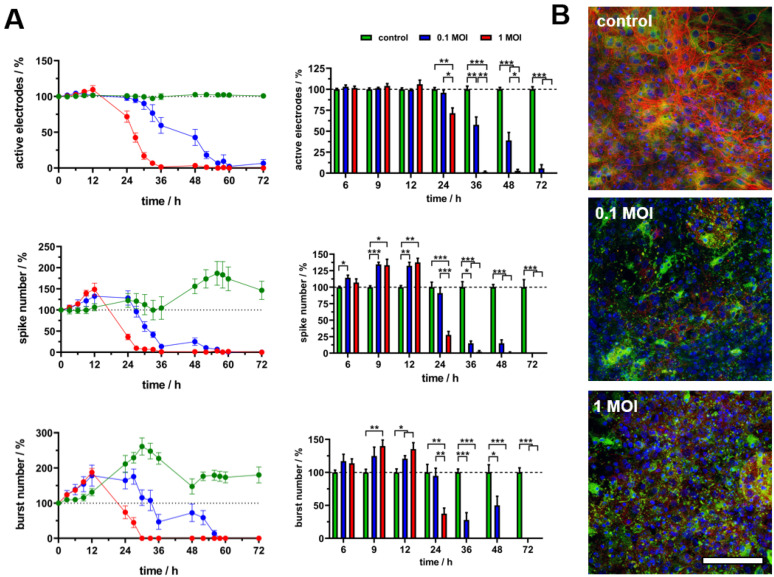
Virus effects on the electrophysiological activity of the neural network. (**A**) Time traces of discrete electrophysiological parameters for control, 0.1 MOI and 1 MOI that are normalised to time point 0 h (**left**) and statistical analysis of values normalised to control (**right**) for selected timepoints (n = 6, mean ± sem, * *p* < 0.05, ** *p* < 0.01, *** *p* < 0.001). (**B**) Immunocytochemical characterisation of neuronal cultures on MEAs after end of experiment (time point 72 h) with neuronal marker neurofilament 200 (red), astrocyte marker GFAP (green) and DAPI nuclei stain (blue) (scale bar 100 µm).

In the next step, the concentration-dependent effect of virus infection on the electrophysiological activity was investigated by field potential monitoring. Therefore, the action potentials were automatically detected for all recorded data and plotted by the self-developed software FiPRAT for an initial review of the overall activity pattern (Supplementary Figures S7–S9). Here, the clear decrease in activity after 48 h (MOI = 0.1) and 24 h (MOI = 1), respectively, was striking. This correlates with the impedimetric monitoring of significant cell layer degeneration, which obviously should also result in a loss of electrophysiological activity. For an in-depth analysis, the recorded raw data of six MEAs per group with cells from three different preparations (n = 6) were analysed regarding several basic descriptive parameters describing the electrophysiological network activity (Figure 4A). First, the number of electrodes with electrophysiological activity were analysed. The relative values per MEA were normalised to time point 0 h (experiment start). The averaged time traces revealed a high stability of electrode numbers with neuronal activity over the whole 72 h, with maximum deviations of 3%. The virus application led to a decrease in electrode numbers with neuronal activity for an MOI of 1 and a more moderate but continuously decreasing number of electrodes for an MOI of 0.1. The statistical analysis for the significant effect of values normalised to the appropriate controls revealed a significant decrease for an MOI of 1 after 24 h and for an MOI of 0.1 after 36 h. For an MOI of 1, the electrode numbers with neuronal activity decreased to almost zero (1.4 ± 0.9 %) after 36 h and did not show any activity in any experiment after 72 h. For an MOI of 0.1, the electrode numbers with neuronal activity decreased to 39.1 ± 9.5 % after 48 h and 5.7 ± 4.5 % after 72 h.

In comparison to the impedimetric-based degeneration of the cell layer, the faster loss of the detectable network activity is comprehensible since this effect of loss of electrophysiologic functionality should clearly happen before a distinct cellular degeneration. For validating the presence of a neural network and the virus-induced breakdown, we fixated the cultures on the MEAs after the experiment (72 h) and stained them for the axonal marker neurofilament 200 (neuronal marker) and astrocyte marker GFAP (Figure 4B). The confocal laser scanning microscope-derived images revealed a pronounced and dense neuronal network with a high amount of long axonal structures as well as a homogeneously distributed support of astrocytes. In contrast, both virus concentrations led to a nearly complete disappearance of the neuronal network, as well as noticeable degeneration of the astrocyte support layer. This strengthens the impedimetric-derived observations for a virus-induced extensive cell layer degeneration as well as the field-potential-derived observation of a complete loss of electrode numbers with neuronal activity.

In the next step, the spike number (number of spikes per minute) was analysed. While after 24 h (MOI of 1) and 36 h (MOI of 0.1), respectively, there was another decrease in the spike number, dependent on virus concentration and time, more strikingly, an initial increase in spike numbers was noticeable. After 6 h, a significant increase could already be detected for an MOI of 0.1 (114.3 ± 3.9%) that further increased up to 134.6 ± 3.3% after 9 h and 132.4 ± 5.4% after 12 h. This effect was comparable for an MOI of 1 (9 h: 133.3 ± 8.9%, 12 h: 137.6 ± 6.2%). The analysis of the burst number (bursts per minute) revealed a comparable effect with a first significant increase after 9 h for an MOI of 1 (139.9 ± 9.2%), and both concentrations after 12 h (MOI of 0.1: 120.8 ± 4.6%, MOI of 1: 135.4 ± 9.7%). This is a very interesting observation and means that there is a distinct virus-induced increase in neuronal activity within the first 12 h after infection, before the virus-caused degenerative process leads to a breakdown of neuronal network activity.

Since there is a lack of comparable in vitro studies on neuronal network activity so far, we found only minor hints for such a virus-induced effect. In a previous study, the effect of the pseudorabies virus on a single rat’s sympathetic neurons was analysed by patch clamp and revealed increased action potential firing rates within 8 h after infection [24].

### 3.5. PrV Kaplan-ΔgG-GFP Virus Leads to Specific Time-Dependent Alteration of Neuronal Network Synchronicity

**Figure 5:**
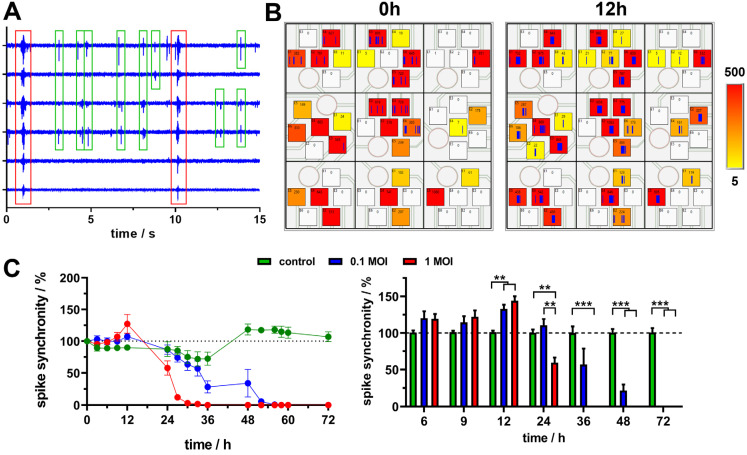
Analysis of neuronal network synchronicity. (**A**) Exemplary field potential traces of neighbouring electrodes with partially (green boxes) and completely (red boxes) synchronised spikes. (**B**) Spatial allocation of spike traces (blue vertical lines in square boxes, box wide corresponds to time window of two seconds) for active electrodes marked as coloured squares (colour code represents spike count per minute with a minimum threshold for activity of 5). Exemplarily selected time points for 1 MOI virus. (**C**) Time traces of spike synchronicity for control, 0.1 MOI and 1 MOI that are normalised to time point 0 h (**left**) and statistical analysis of values normalised to control (**right**) for selected time points (n = 6, mean ± sem, ** *p* < 0.01, *** *p* < 0.001).

Beyond the analysis of individual electrode time traces and, thereof, derived activity parameters, the big advantage of microelectrode arrays is the clearly defined spatial resolution that allows the identification and analysis of network interactions. For demonstration, the field potential traces of six selected neighbouring electrodes were used (Figure 5A) to show partial and complete coordinated network communication, which can be described as synchronous signalling events.

Based on the knowledge of the electrode position, the activity can be spatially matched (Figure 5B zoom view, Supplementary Figures S10 and S11). The exemplarily shown maps for an MOI of 1 at 0 h and 12 h reveal a higher number of synchronously occurring spikes for 12 h, which means a higher network synchronicity. For a quantitative analysis of network synchronicity, the recorded raw data of six MEAs from three experiments were analysed. The time traces normalised to 0 h (Figure 5C) revealed a comparable effect to those observed for the spike and burst numbers. The virus application led to an initial significant increase in the synchronicity after 12 h (MOI of 0.1: 132.6 ± 13.5%, MOI of 1: 143.9 ± 13.9%), followed by a significant decrease after 24 h (MOI of 1) and 48 h (MOI of 0.1), respectively. While the later occurring decrease in network synchronicity comprehensibly correlates with the virus-induced degeneration of the network, the initially observed increase is, again, a very interesting observation. This means that the virus not only causes a temporal increase in neuronal activity, but also an increased synchronous network signalling.

## 4. Conclusions

In the presented study, we were able to demonstrate the successful development of a hybrid measurement system that is capable of high-resolved impedance spectroscopy and field potential monitoring on the same microelectrode. By using specific low noise switches for both measurement pathways, even high intrinsic impedance of small microelectrode measurement artefacts and high noise levels could be suppressed. Although there already is a developed field potential monitoring system described with, e.g., a much higher number of electrodes/electrode densities (e.g., CMOS MEAs), there is no other system described that offers the demonstrated capabilities and flexibilities for hybrid monitoring on microelectrode arrays (Supplementary Table S1). Moreover, the experiments were carried out using two 60-channel hybrid frontends in parallel to demonstrate the capabilities for parallelisation of the system. The current system can be easily scaled up to six parallel frontends for a more comfortable analysis of different experiment conditions. Moreover, the DAQ system is designed for further upscaling. Thus, thousands of measurement channels could be easily realised on demand.

For a demonstration of the system’s capabilities, the pseudorabies model virus PrV Kaplan-ΔgG-GFP was applied on hippocampal cultures. The analysis revealed a structural and functional degeneration of the neuronal network within 24 to 48 h. More strikingly, we could detect a temporal increase in neuronal activity as well as synchronicity within the first 12 h after virus application. Therefore, this demonstrates an advantage for the complementary read-out of information on the same electrode and the same cell sample, in the sense of a multimodal monitoring in order to draw synergistic conclusions about the behaviour or reaction of neuronal networks to neuron-infecting viruses. Based on this successful proof of principle, the developed system can be used for the detailed long-term analysis of, e.g., the rabies virus (RABV) on different target hosts’ in vitro models. Moreover, the multimodal hybrid monitoring platform could even be used for the analysis of complex virus effects in human neuronal networks that could be generated, e.g., from hiPSC.

Such in vitro systems could help to answer urgent medical questions of potential neurological risks from novel occurring viruses like coronavirus SARS-CoV-2.

## Data Availability

Data is contained within the article or Supplementary Material, raw dataset is available on request from the authors.

## Supplementary Materials

The following supporting information can be downloaded at: https://www.mdpi.com/article/10.3390/bios14060295/s1, References [25,26,27,28] are cited in the Supplementary Materials.
