# Supplementary material for: Novel Multiparametric Bioelectronic Measurement System for Monitoring Virus-Induced Alterations in Functional Neuronal Networks
Source: Biosensors (Basel). 2024 Jun 5;14(6):295. doi: 10.3390/bios14060295 (PMC11202209; doi:10.3390/bios14060295)
Supplement: Supplementary file 1 [file biosensors-14-00295-s001.zip › biosensors-3003092-supplementary.pdf]

Supplementary Materials

Novel Multiparametric Bioelectronic Measurement System for Monitoring Virus-Induced Alterations in Functional Neuronal Networks

Heinz-Georg Jahnke [1,*,†], Verena te Kamp [2,†], Christoph Prönnecke [1], Sabine Schmidt [1], Ronny Azendorf [1], Barbara Klupp [2], Andrea A. Robitzki [3] and Stefan Finke [2]

[1] Centre for Biotechnology and Biomedicine, Biochemical Cell Technology, Leipzig University, Deutscher Platz 5, 04103 Leipzig, Germany; sabine.schmidt@bbz.uni-leipzig.de (S.S.)
[2] Institute of Molecular Virology and Cell Biology, Friedrich-Loeffler-Institut, Südufer 10, 17493 Greifswald, Germany; barbara.klupp@fli.de (B.K.)
[3] Division Management for Biology, Chemistry and Process Engineering, Karlsruhe Institute of Technology (KIT), Hermann-von-Helmholtz-Platz 1, 76344 Eggenstein-Leopoldshafen, Germany
* Correspondence: heinz-georg.jahnke@bbz.uni-leipzig.de; Tel.: +49-341-973-1246; Fax: +49-341-973-1249
† These authors contributed equally to this work.

Supplementary Table

Table S1: Comparison of microelectrode array based measurement systems for the (multimodal) bioelectronic analysis of neuronal network cell culture models.

		(Miccoli et al. 2019)	(Emery et al. 2023)	(Zhao et al. 2023)	(Middya et al. 2021)	(Hu et al, 2023)	present work
MEA	**type**	active (CMOS)	active (CMOS)	active CMOS + passive flex2chip	passive	passive	passive
	**number and type of electrodes**	4,096 pixels with 4 microelec. / pixel (8,8-121 µm²)	4,096 microelec. (441 µm²)	26,400 / 2,200 microelec. (314 µm²)	60 -120 microelec. (700 µm²)	2 microelec. (7850 µm²)	512 microelectrodes (700-7850 µm²)
	**parallel read-out channels**	1,024	4,096	1,024	60 - 120	2	54 x 6 (324)
	**substrate**	silicone (opaque)	silicone (opaque)	silicone / polyimide foil	glass (highly transparent)	glass (highly transparent)	glass (highly transparent)
	**complexity / flexibility / fabrication expense**	high / low / high	high / low / high	high / low / high	low / high / low	low / high / low	low / high / low
platform	**DAQ-system**	self-developed	3Brain AG	MaxWell Biosystems + self-developed	Multichannel Systems MCS GmbH	Axon Multiclamp 700B amplifier (Molecular Devices).	Sciospec Scientific Instruments GmbH
	**signal-to noise ratio (FPM)**	low	low	low	high	high	high
	**EIS**	basic function (single frequency 1kHz) for cell coverage detection	-	-	-	only between 2 large area finger-like electrodes, no spatial resolution	high-precision with high spatial and time resolution mode
	**compatible with optical monitoring**	-	-	-	high-resolution microscopy	high-resolution microscopy	high-resolution microscopy
	**validated cell types**	neuronal networks	neuronal networks	neuronal networks (in vivo)	neuronal networks	cardiomyocytes	neuronal networks
	long-term monitoring / viability	short-term	short-term	short-term	short-term	short-term	> 30 days

Supplementary Figures

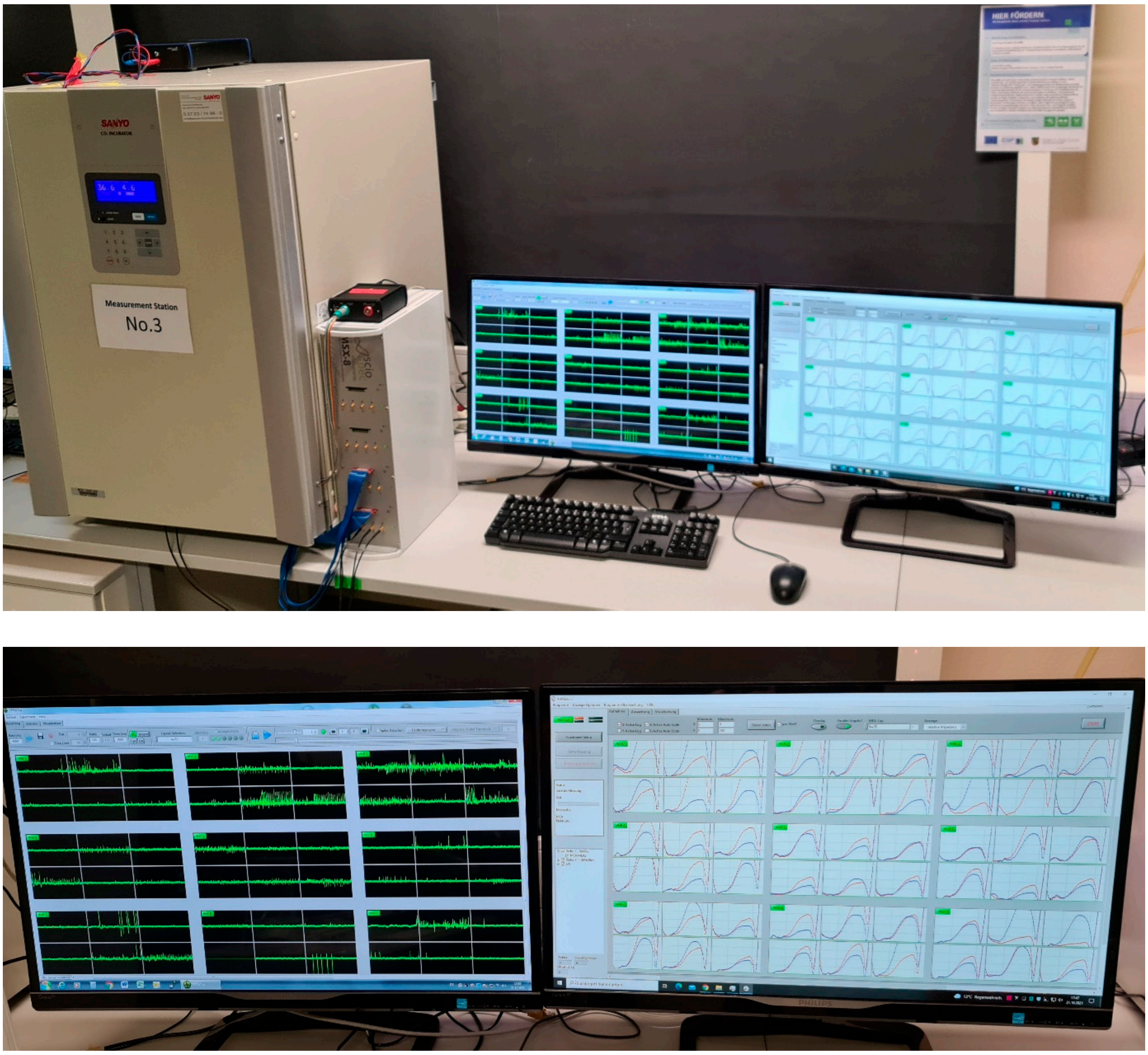

Figure S1: Hybrid measurement setup and controlling Software GUI.

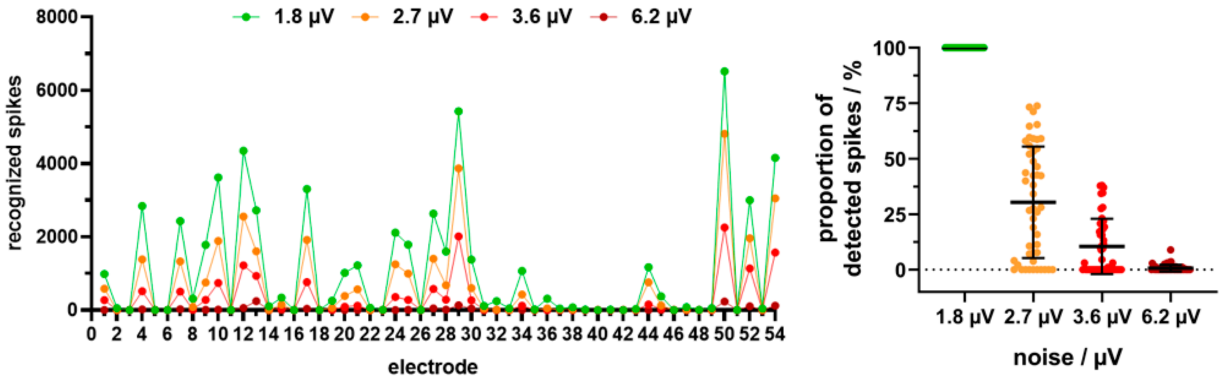

Figure S2: Analysis of microelectrode size dependent noise level on the number of detectable spikes. Detected spikes for each single electrode of a MEA based on different noise levels (left). Proportion of detected spikes in comparison to a noise level of 1.8 µV (right) (n = 48, mean ± sd).

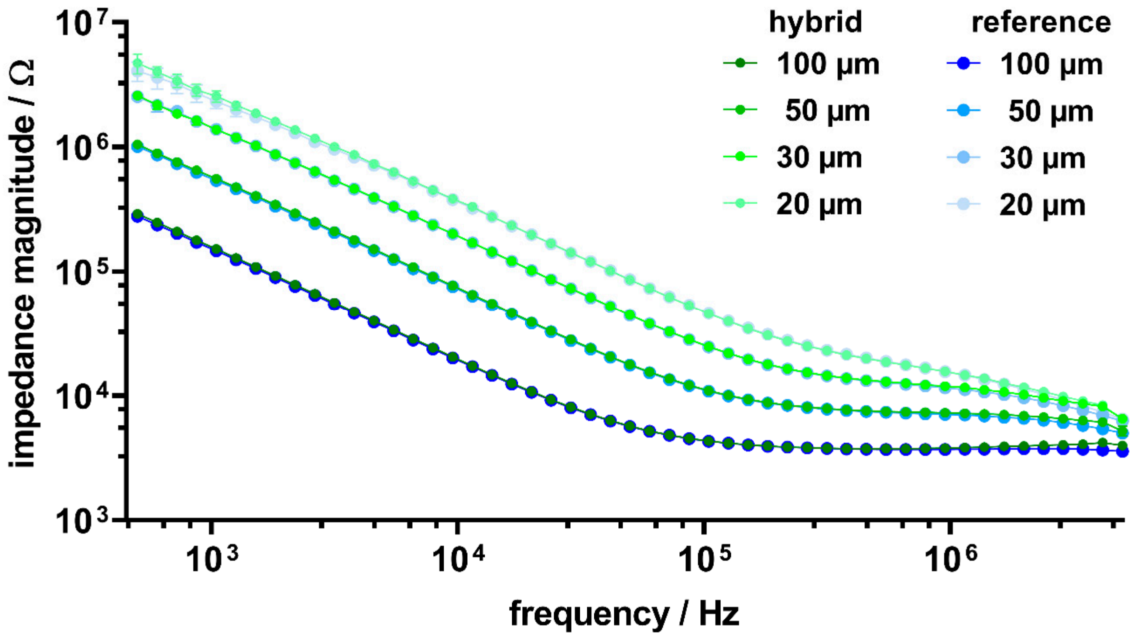

Figure S3: Impedance magnitude spectra. Comparison of electrode size (diameter) dependent impedance magnitude spectra for only an impedance reference frontend and the novel hybrid frontend (n = 8, mean ± sd).

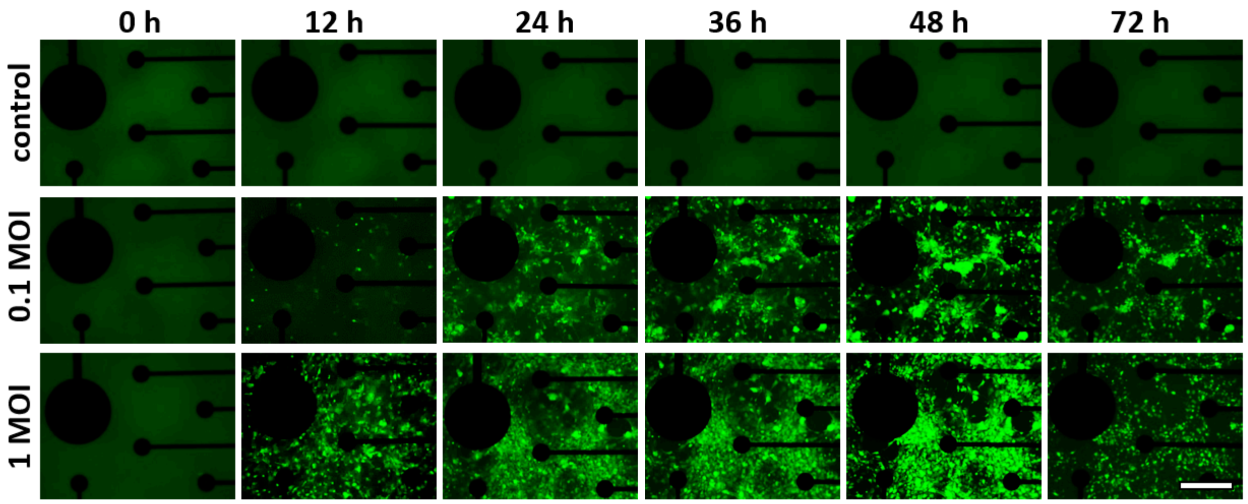

Figure S4: Fluorescence microscopic images for tracing virus infection. Cell infection was visualized by the GFP labeled virus particle that could be traced over the whole experiment duration. (scale bar = 400 µm)

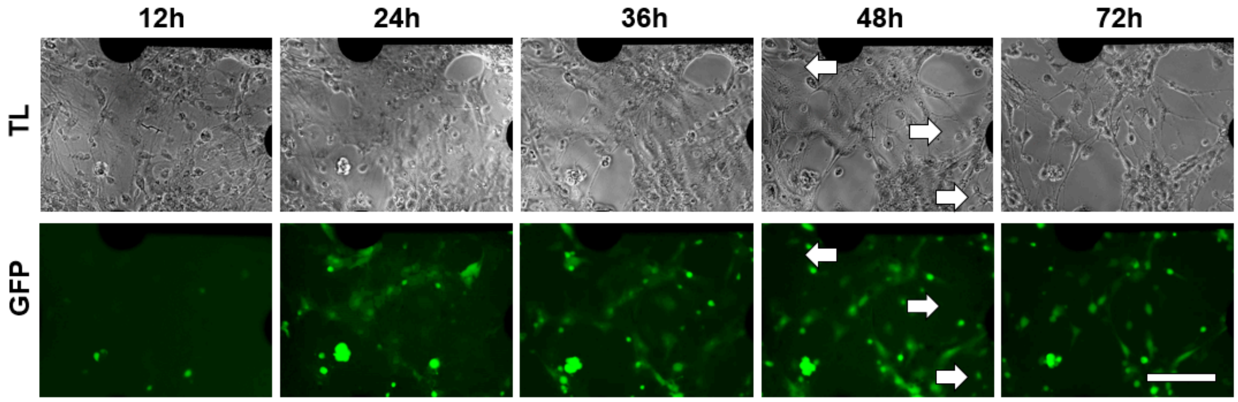

Figure S5: Microscopic monitoring of virus induced morphological degeneration of neuronal networks. Transmitted light (TL) and fluorescence (GFP) microscopic images of neuronal cultures on an exemplarily MEA incubated with a virus MOI of 0.1 at discrete time points. White arrows mark initial areas of morphological dissolution / degeneration of the cell layer (scale bar = 200 µm)

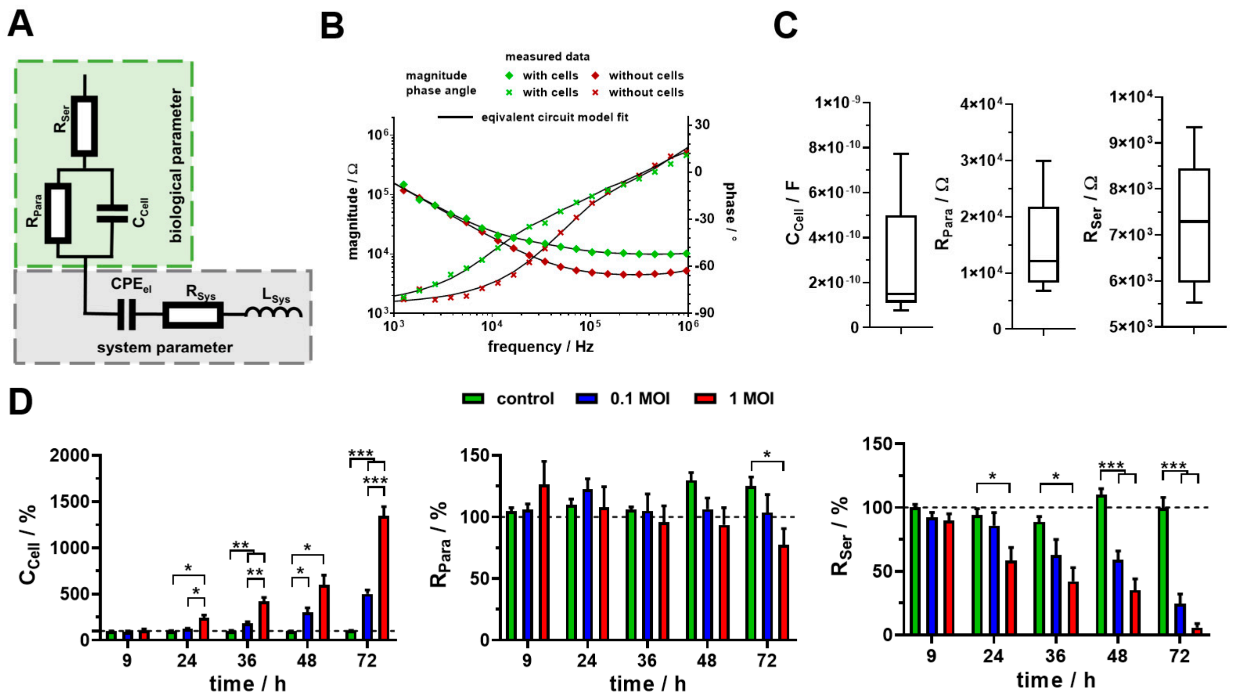

Figure S6: Electrical equivalent circuit model based impedance data analysis. (A) Scheme of applied electrical equivalent circuit model (EECM) for 2-step parameter fitting of system parameter (first step) and biological parameter (second step). (B) Example of measured data and EECM derived magnitude and phase angle spectra. (C) Statistical analysis of determined biological parameters C_{Cell}, R_{Para} and R_{Ser} for 18 MEAs (box blot with quartiles and min/max whiskers). (D) Statistical analysis of biological parameters normalized to time point 0 h, ($n = 6$, mean ± sem, $^{*}p < 0.05$, $^{**}p < 0.01$, $^{***}p < 0.001$).

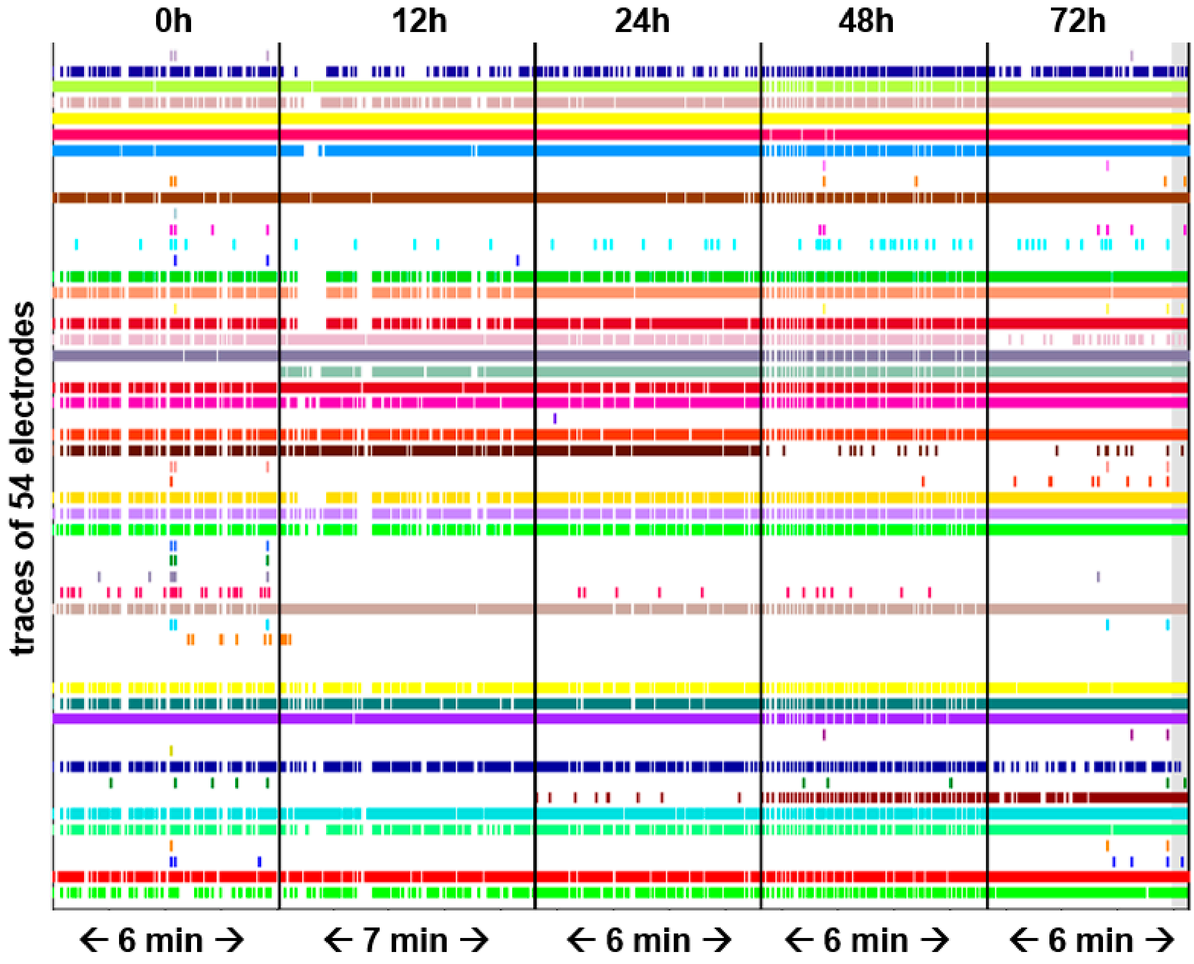

Figure S7: Exemplarily trace of detected action potentials for a single MEA (control) at selected time points.

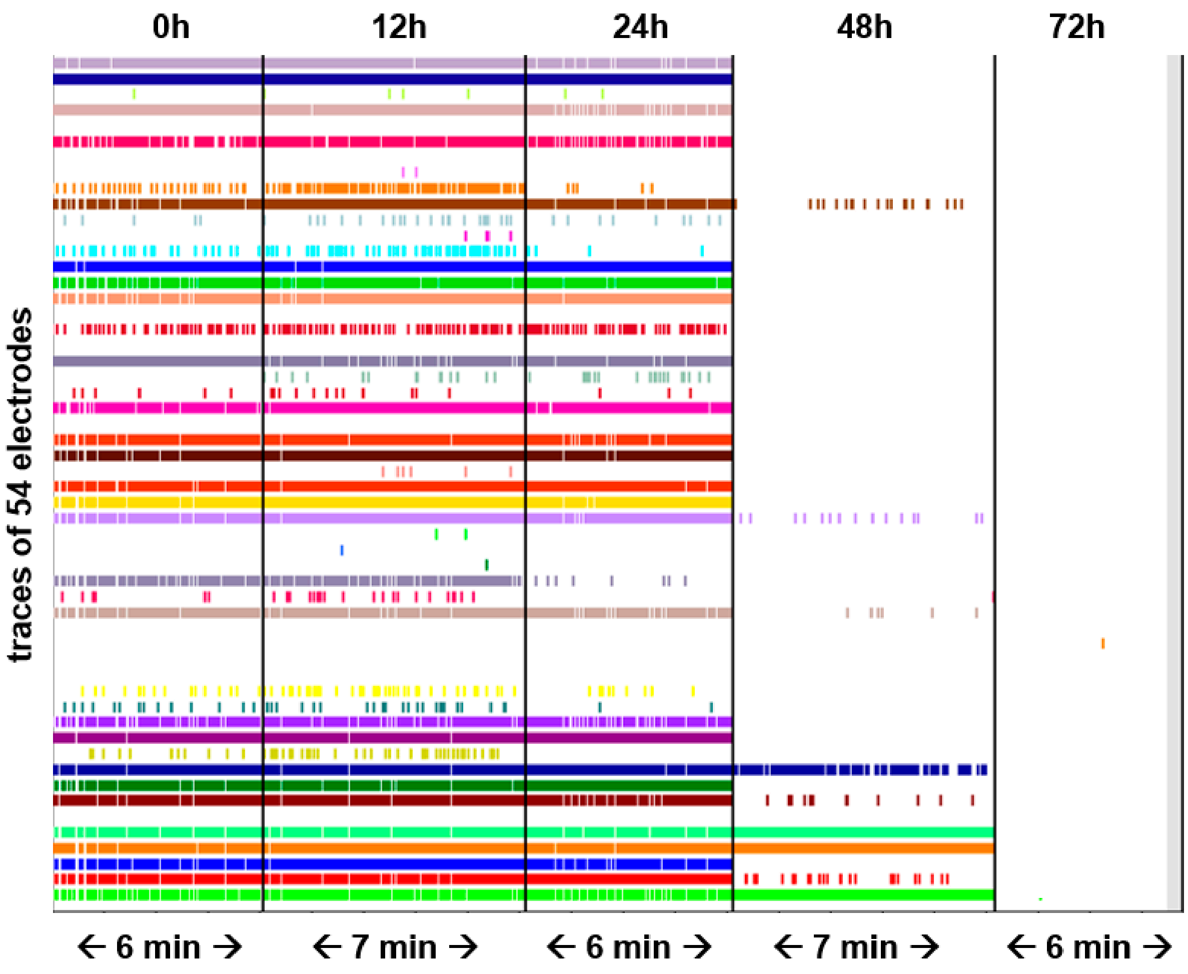

Figure S8: Exemplarily trace of detected action potentials for a single MEA (MOI of 0.1) at selected time points.

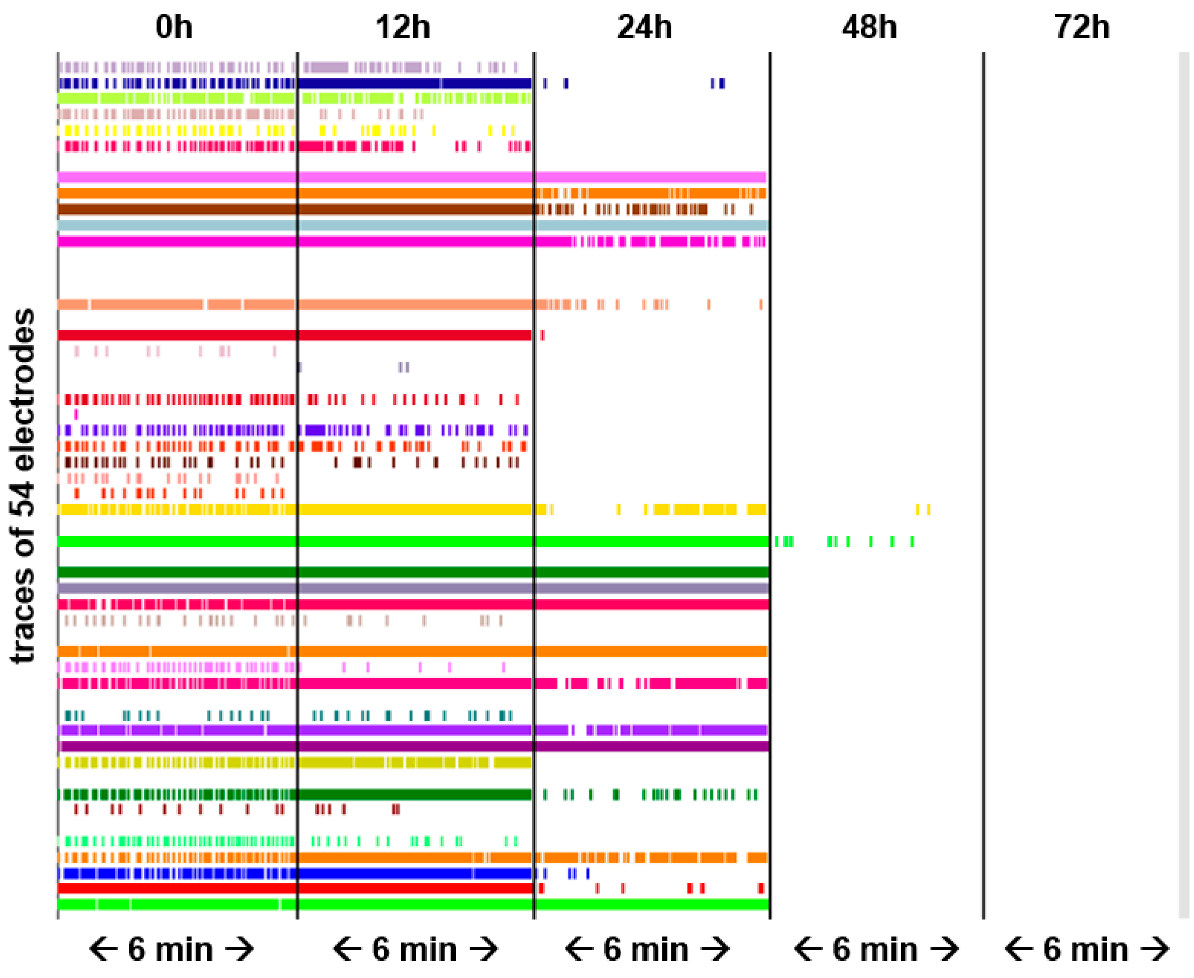

Figure S9: Exemplarily trace of detected action potentials for a single MEA (MOI of 1) at selected time points.

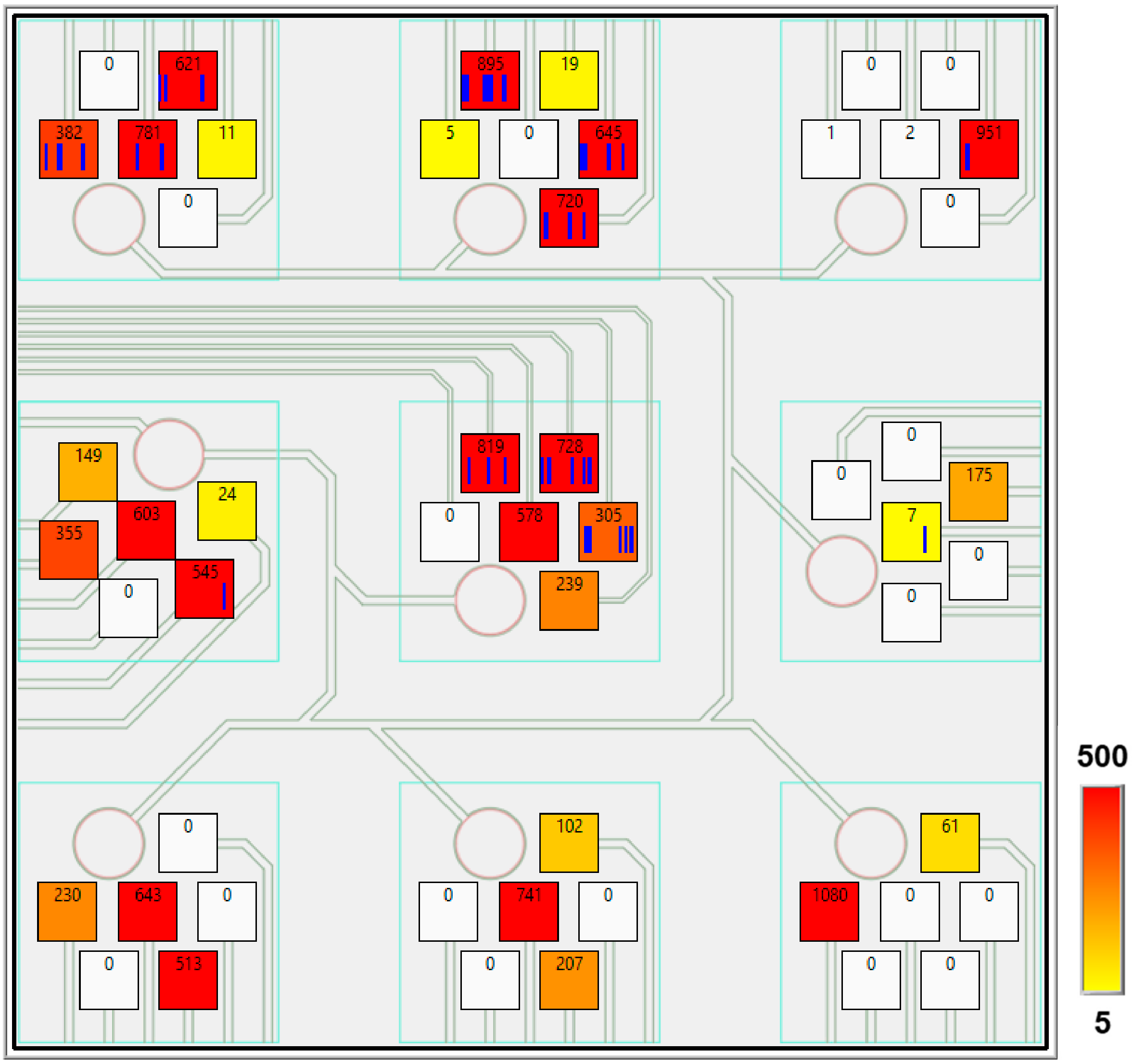

Figure S10: Exemplarily demonstration of spatial allocation of spike traces for a MOI of 1 at 0 hours. Detected spikes are represented as vertical blue lines. The scare as representation of electrodes depicts a time schedule of two seconds. Active electrodes (squares) are colored. The color code represents spike count per minute with a minimum threshold for activity of 5). The appropriate value is also shown in the squares.

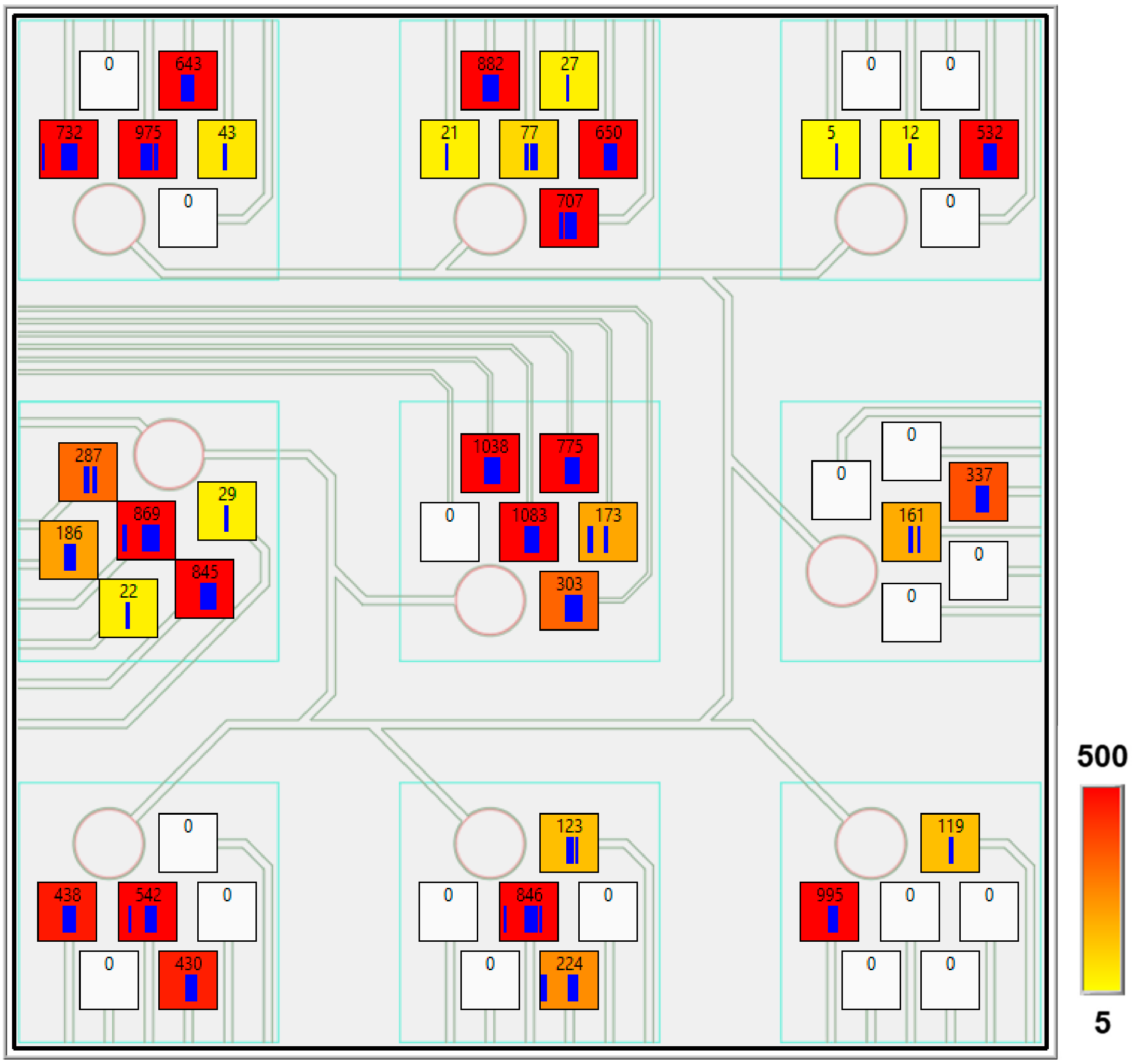

Figure S11: Exemplarily demonstration of spatial allocation of spike traces for a MOI of 1 at 12 hours. Detected spikes are represented as vertical blue lines. The scare as representation of electrodes depicts a time schedule of two seconds. Active electrodes (squares) are colored. The color code represents spike count per minute with a minimum threshold for activity of 5). The appropriate value is also shown in the squares.

References

Emery, B.A., Hu, X., Khanzada, S., Kempermann, G., Amin, H., 2023. High-resolution CMOS-based biosensor for assessing hippocampal circuit dynamics in experience-dependent plasticity. Biosens. Bioelectron. 237, 115471.

Miccoli, B., Lopez, C.M., Goikoetxea, E., Putzeys, J., Sekeri, M., Krylychkina, O., Chang, S.W., Firrincieli, A., Andrei, A., Reumers, V., Braeken, D., 2019. High-Density Electrical Recording and Impedance Imaging With a Multi-Modal CMOS Multi-Electrode Array Chip. Front. Neurosci. 13, 641.

Middya, S., Curto, V.F., Fernandez-Villegas, A., Robbins, M., Gurke, J., Moonen, E.J.M., Kaminski Schierle, G.S., Malliaras, G.G., 2021. Microelectrode Arrays for Simultaneous Electrophysiology and Advanced Optical Microscopy. Adv Sci (Weinh) 8(13), 2004434.

Zhao, E.T., Hull, J.M., Mintz Hemed, N., Ulusan, H., Bartram, J., Zhang, A., Wang, P., Pham, A., Ronchi, S., Huguenard, J.R., Hierlemann, A., Melosh, N.A., 2023. A CMOS-based highly scalable flexible neural electrode interface. Sci Adv 9(23), eadf9524.
